# Acute Bioprosthetic Mitral Valve Failure Diagnosed Using Point-of-Care Ultrasound Leading to Prompt Treatment and Good Outcome

**DOI:** 10.1016/j.case.2022.05.004

**Published:** 2022-08-15

**Authors:** Pyi Naing, Katherine Lau, Paul Wiemers, Andrew Mulligan, Matthew K. Burrage, Gregory M. Scalia

**Affiliations:** a Prince Charles Hospital, Chermside, Queensland, Australia; b University of Notre Dame, Fremantle, Western Australia, Australia; c University of Queensland, Brisbane, Queensland, Australia

**Keywords:** POCUS, Acute mitral regurgitation, Bioprosthetic valve failure, Mitral valve-in-valve replacement

## Abstract

• Acute bioprosthetic mitral valve failure is a medical emergency.
• POCUS can confirm the diagnosis along with a good history and physical exam.
• Early diagnosis and prompt multidisciplinary treatment will deliver the best outcome.

## Graphical abstract

**Figure:**
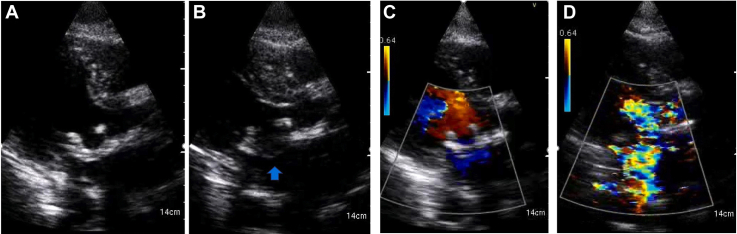

## Introduction

Acute severe mitral regurgitation (MR) is a medical emergency that requires prompt diagnosis and treatment.^1^ Typical clinical signs include a pansystolic murmur at the cardiac apex and pulmonary congestion. Failure to recognize these clinical clues can lead to delayed diagnosis and treatment, resulting in poor outcomes. Definitive diagnosis requires echocardiography. We present a patient with bioprosthetic valve failure and acute severe MR whose clinical course was significantly impacted by prompt diagnosis using point-of-care ultrasound (POCUS), enabling timely life-saving expert multidisciplinary care.

## Case Presentation

A 79-year-old woman presented to our emergency department with a 24-hour history of acute severe dyspnea. Her medical history was significant for long-standing ankylosing spondylitis treated with methotrexate and adalimumab. She had previously undergone 2 cardiac valve replacement surgeries. A combined Trifecta 19 mm bioprosthetic aortic valve replacement and Mosaic 27 mm bioprosthetic mitral valve replacement (MVR) had been performed 6 years prior for degenerative aortic and MR. She subsequently underwent redo aortic valve surgery (Perimount 19 mm; Carpentier-Edwards) for severe degenerative calcific prosthetic valve stenosis only 1 month prior to her current presentation. Periprocedurally there had been no significant MR noted, and bioprosthetic mitral valve hemodynamics were within normal ranges. The mitral valve was directly inspected at the time of operation with no macroscopic abnormality noted.

A provisional diagnosis of congestive cardiac failure was made by the emergency medical team, and the patient was admitted directly to the general cardiology ward. On review by the on-call cardiology team after hours, she was found to be tachypneic with increased work of breathing and increasing fatigue. She was also hypotensive (blood pressure, 101/65 mm Hg), borderline tachycardiac (heart rate, 96 beats per minute), and hypoxic (peripheral oxygen saturation by pulse oximetry was 90% on room air). Auscultation revealed a loud pansystolic murmur radiating to the axilla and back. Chest auscultation revealed widespread crepitations consistent with pulmonary edema.

**Figure 1:**
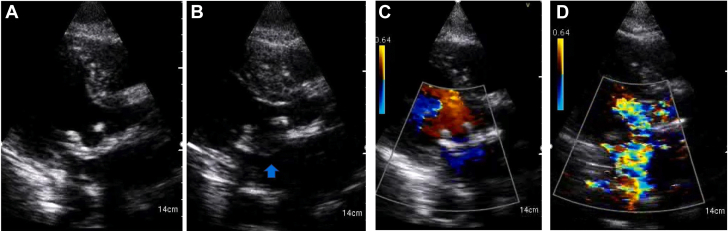
POCUS images showing bioprosthetic mitral valve in diastole **(A)**, flail leaflet of mitral valve prothesis (*blue arrow* in panel **B**), color Doppler image diastole **(C)**, and severe MR on color Doppler imaging **(D)** during systole.

**Figure 2:**
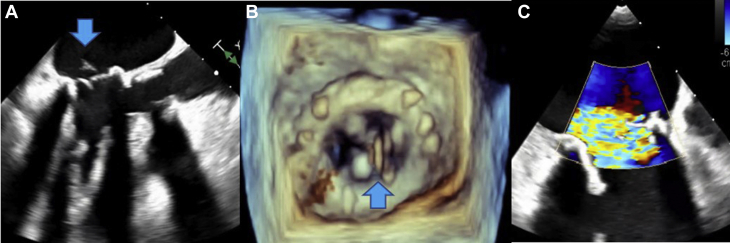
**(A, B)** Intraprocedural TEE confirming the flail mitral valve leaflet (*blue arrows*) causing severe MR **(C)**.

**Figure 3:**
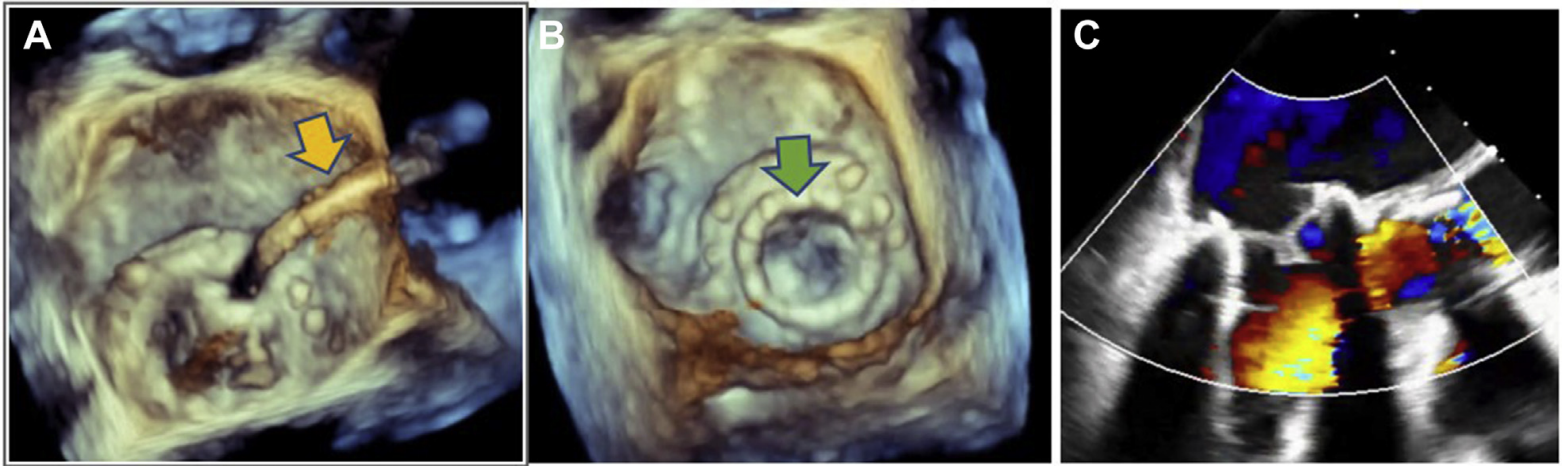
Transesophageal echocardiogram showing the new transcatheter mitral valve-in-valve transseptal delivery system (**A**, *yellow arrow*) and replacement transcatheter heart valve (**B**, *green arrow*). **(C)** New competent mitral valve without regurgitation.

Emergency POCUS study using a handheld device was performed at the patient's bedside in the general cardiology ward because conventional echocardiography was not readily available after hours. This revealed hyperdynamic left ventricular systolic function and a well-seated bioprosthetic MVR. There was severe MR with a flail leaflet seen on the atrial aspect of the MVR (Figure 1, Videos 1 and 2). The patient was transferred to the coronary care unit for further management and stabilization, including use of intravenous diuretics and nitrates for acute pulmonary edema. Based on the POCUS findings, an emergency heart team meeting was convened consisting of noninvasive cardiologists, interventional structural cardiologists, and cardiac surgeons, with a plan to proceed with semiemergent percutaneous mitral valve-in-valve replacement. Despite initial stabilization, the patient continued to deteriorate on medical therapy and required an emergency mitral valve-in-valve procedure within 24 hours. The intraprocedural transesophageal echocardiogram (TEE) confirmed the POCUS findings with a flail bioprosthetic mitral valve leaflet and severe MR (Figure 2, Videos 3 and 4). The patient experienced a rapid recovery following the successful deployment of a transcatheter heart valve (Figure 3, Video 5, Video 6, Video 7) and was discharged home 5 days later. All blood cultures were negative for microorganisms. She continued to improve and was asymptomatic at clinic follow-up 3 months postprocedure.

## Discussion

Acute MR should be suspected in patients who present with acute-onset dyspnea, especially in those with a history of mitral valve surgery. Although a pansystolic murmur is commonly heard in MR, the murmur may be soft or short in acute presentations due to rapid pressure equalization. The acute pressure and volume overload on a small, noncompliant left atrium and pulmonary circulation will lead to pulmonary edema.

POCUS is an increasingly popular method to aid in the diagnosis of patients presenting with acute dyspnea. The benefits of POCUS include the portability, speed, and ability to perform imaging at the patient's bedside.^2^ During the COVID-19 pandemic, POCUS has also proven very useful as the portable ultrasound machines are easier to clean and sterilize.^3^ The POCUS examination should be targeted based on presenting symptoms and clinical signs. The comprehensive echocardiography should always be performed when the patient is more stable as POCUS may miss some subtle but important pathologies. Cardiovascular POCUS can provide important information regarding left ventricular systolic function, valvular dysfunction, and pericardial tamponade. In our patient, POCUS has proven its usefulness by revealing the exact pathology of bioprosthetic mitral valve dysfunction in a timely manner. Our patient presented when comprehensive formal echocardiography was not readily available. As such, the complete transthoracic echocardiogram could not have been performed until more than a day later, which would have led to a delayed diagnosis and a potentially unfavorable outcome. The findings from POCUS enabled an early diagnosis and facilitated the necessary preparation by the expert multidisciplinary team, allowing for prompt treatment. In institutions where the after-hours availability of full echocardiography machines is limited such as in our hospital, POCUS can be very valuable in the diagnosis of acute cardiac pathologies in addition to thorough clinical assessment.

Bioprosthetic heart valves are increasingly more popular than mechanical heart valves owing to their superior hemodynamics, reduced thrombogenicity, and the availability of less invasive transcatheter percutaneous valve interventions.^4^^,^^5^ However, bioprosthetic heart valves are less durable and may require further surgery or reintervention later in life. Mechanisms of bioprosthetic valve failure are not fully understood but can be due to calcification, infection, thrombus formation, immune responses, and mechanical degeneration.^4^^,^^6^ Interestingly, our patient's MVR was examined during the aortic valve surgery 1 month prior and deemed to be functioning normally. The cause of acute valvular deterioration in this patient was unclear and was not found to be due to acute infective endocarditis.

There are good data to support the use of transcatheter valves to replace failed bioprosthetic aortic valves^7^, ^8^, ^9^, ^10^; however, the data are still limited for the use of transcatheter valves for failed bioprosthetic mitral valves.^11^^,^^12^ There are also concerns for left ventricular outflow obstruction caused by the transcatheter MVR, with low-profile valves and newer valve designs being used to avoid this. In our patient, the percutaneous valve-in-valve replacement was a more attractive option as she recently had undergone a second sternotomy for a redo heart valve replacement surgery. Our patient achieved a good outcome owing to the prompt diagnosis with emergency POCUS as well as timely treatment by the expert multidisciplinary team.

## Conclusion

This case highlights the importance of thorough clinical examination and the added benefit of POCUS to make a prompt life-saving diagnosis of acute bioprosthetic valve degeneration at the patient's bedside allowing emergency definitive treatment. POCUS is an important adjunctive tool to assess patients, alongside history taking and clinical examination, when there is a high index of suspicion for acute reversible cardiac pathologies.

## Supplementary Data

Video 1 Two-dimensional POCUS transthoracic echocardiogram parasternal long-axis view demonstrates bioprosthetic mitral valve with flail leaflet seen on the atrial aspect.

Video 2 POCUS transthoracic echocardiogram parasternal long-axis view with color Doppler demonstrates severe bioprosthetic MR.

Video 3 Intraprocedural three-dimensional TEE demonstrates flail bioprosthetic mitral valve leaflet.

Video 4 Intraprocedural two-dimensional biplane TEE demonstrates severe bioprosthetic MR.

Video 5 Intraprocedural three-dimensional TEE demonstrates deployment of new transcatheter heart valve using transeptal approach.

Video 6 Intraprocedural three-dimensional TEE demonstrates successful deployment of transcatheter heart valve.

Video 7 Intraprocedural two-dimensional color Doppler TEE demonstrates that no MR is detected following the new transcatheter mitral valve.
